# Assessment of quality of life in small-cell lung cancer using a Daily Diary Card developed by the Medical Research Council Lung Cancer Working Party.

**DOI:** 10.1038/bjc.1991.296

**Published:** 1991-08

**Authors:** P. M. Fayers, N. M. Bleehen, D. J. Girling, R. J. Stephens

**Affiliations:** Cancer Trials Office, Cambridge, UK.

## Abstract

Three hundred and sixty-nine patients in an MRC study of combination chemotherapy and radiotherapy for small-cell lung cancer of limited extent were asked to complete a Daily Diary Card which enabled an assessment of their quality of life to be made during and after treatment. The information derived from the card suggests that although cytotoxic chemotherapy has an adverse effect upon quality of life, this impairment only affects the first 2 or 3 days following each course of treatment, although there is also a small deterioration which may be associated with the 'nadir' effect of the blood counts about 10 days after each course. These results should assist physicians in counselling patients about the likely effects of treatment. Just over half of the patients (196) were subsequently randomised to either a further six courses of maintenance chemotherapy or no further chemotherapy, and it is also shown that the patients allocated to no maintenance chemotherapy experienced a gradually deteriorating quality of life, as opposed to the brief but more severe adverse effects which occurred following each course in the maintenance chemotherapy group; this supports the hypothesis of a palliative effect in this latter group. The findings demonstrate that the Daily Diary Card is a sensitive instrument capable of yielding useful information.


*Br. J. Cancer* (1991), **64**, 299–306 © Macmillan Press Ltd., 1991

### Assessment of quality of life in small-cell lung cancer using a Daily Diary Card developed by the Medical Research Council Lung Cancer Working Party

Report to the Medical Research Council by its Lung Cancer Working Party
Prepared on behalf of the participating members by

P.M. Fayers (Statistician), N.M. Bleehen (Chairman until October 1989), D.J. Girling (Secretary) & R.J. Stephens (Data Manager)

**Summary** Three hundred and sixty-nine patients in an MRC study of combination chemotherapy and radiotherapy for small-cell lung cancer of limited extent were asked to complete a Daily Diary Card which enabled an assessment of their quality of life to be made during and after treatment. The information derived from the card suggests that although cytotoxic chemotherapy has an adverse effect upon quality of life, this impairment only affects the first 2 or 3 days following each course of treatment, although there is also a small deterioration which may be associated with the 'nadir' effect of the blood counts about 10 days after each course. These results should assist physicians in counselling patients about the likely effects of treatment. Just over half of the patients (196) were subsequently randomised to either a further six courses of maintenance chemotherapy or no further chemotherapy, and it is also shown that the patients allocated to no maintenance chemotherapy experienced a gradually deteriorating quality of life, as opposed to the brief but more severe adverse effects which occurred following each course in the maintenance chemotherapy group; this supports the hypothesis of a palliative effect in this latter group. The findings demonstrate that the Daily Diary Card is a sensitive instrument capable of yielding useful information.

Small-cell lung cancer carries a poor prognosis but is usually highly sensitive to cytoxic chemotherapy and radiotherapy. Such treatment policies aim to control the symptoms of the disease, prolong survival, and will cure a small proportion of the patients. However, although the primary disease and its metastases may be controlled, and therefore the symptoms reduced, the adverse effects of treatment may be unpleasant, and all too often there is little gain in the number of long term survivors. A Medical Research Council (MRC) study of chemotherapy in the treatment of small-cell lung cancer attempted to assess this (MRC Lung Cancer Working Party, 1989). Since long term survival is uncommon, the patients' quality of life whilst receiving treatment is important. In particular, since the chemotherapy may be toxic it is desirable to limit its duration to the shortest that can achieve maximum benefit. These considerations have led to an increased interest in methods of assessing quality of life, and they are now frequently included in clinical trials. Such assessments may be made by the clinician, a nurse, the patient or a relative; usually, however, they only report a summary of the patient's quality of life since the previous attendance at the hospital or over some similarly long time span. In earlier studies the MRC Lung Cancer Working Party had used standard scales applied by clinicians at such intervals to assess performance status and general health. In this study it was decided to develop a Diary Card to be completed by patients on a daily basis (Figure 1) so as to examine the way in which the patient's general health varies during and after a course of treatment. The results from this Daily Diary Card are presented, and the problems associated with developing and applying new instruments for assessing quality of life are discussed. Similar cards are now being used in a number of current MRC studies, and by other groups (Geddes *et al.*, 1990).

Correspondence: P.M. Fayers, M.R.C., Cancer Trials Office, 1 Brooklands Avenue, Cambridge CB2 2BB, UK.
Received 9 March 1990; and in revised form 25 March 1991.

### Methods

### *Patients and outline of study*

The design of the study is reported in greater detail elsewhere (MRC Lung Cancer Working Party, 1989). In this paper only those study patients with limited disease are described, since the protocol treatment for patients with extensive disease was different. In essence, patients with small-cell lung cancer were eligible for the study if they were aged 75 years or less, and had 'good performance status', that is were able to get out and about even if activity was restricted. An initial induction period of treatment of six courses of chemotherapy was given, with radiotherapy between courses two and three. Patients who were responding to treatment were then randomly assigned to a policy of maintenance (M) or no maintenance (NoM) chemotherapy. Since the benefits of treatment in terms of survival were uncertain, it was considered important to investigate the quality of life during the M/NoM period in order to compare the possible gain in survival duration against the adverse effects of continued treatment.

### *Details of treatment policies*

All patients were given induction treatment with six courses of cytotoxic chemotherapy; this comprised chemotherapy on 3 consecutive days: etoposide 120 mg $m^{-2}$, cyclophosphamide 1 g $m^{-2}$, methotrexate 35 mg $m^{-2}$, and vincristine 1.3 mg $m^{-2}$ intravenously on day 1, then etoposide 240 mg $m^{-2}$ orally or 120 mg $m^{-2}$ intravenously on days 2 and 3. Courses were given at 3-week intervals. In addition, megavoltage radiotherapy was given over 3 weeks between the 2nd and 3rd courses of chemotherapy, starting 3 weeks after the second course. A radiotherapy midline dose of 40 Gy in 15 daily fractions 5 days per week over 3 weeks was delivered through planned portals to the primary site and mediastinal lymph nodes. At the end of the initial induction period of chemotherapy patients showing complete or partial response to treatment (World Health Organization, 1979) were allocated at random to receive either no further treatment (No Maintenance – 'NoM') or a further six courses of the same

Hospital number 012345

Doctor in charge Dr J. Smith

Date of start of this card 1 April 1988

| | WEEK 1 | | | | | | | WEEK 2 | | | | | | | WEEK 3 | | | | | | |
|---|---|---|---|---|---|---|---|---|---|---|---|---|---|---|---|---|---|---|---|---|---|
| | Mon | Tue | Wed | Thu | Fri | Sat | Sun | Mon | Tue | Wed | Thu | Fri | Sat | Sun | Mon | Tue | Wed | Thu | Fri | Sat | Sun |
| SICKNESS (VOMITING) | | | 1 | 4 | 3 | 2 | 1 | 1 | 1 | 3 | 2 | 2 | 2 | 1 | 2 | 2 | 2 | 2 | 2 | 1 | 1 |
| ANXIETY | | | 4 | 4 | 5 | 4 | 2 | 2 | 3 | 3 | 2 | 2 | 2 | 3 | 3 | 2 | 2 | 2 | 3 | 3 | 2 |
| MOOD | | | 2 | 3 | 5 | 4 | 3 | 4 | 3 | 3 | 2 | 2 | 2 | 3 | 2 | 2 | 3 | 4 | 3 | 2 | 2 |
| ACTIVITY | | | 2 | 2 | 4 | 4 | 3 | 4 | 4 | 3 | 2 | 2 | 2 | 3 | 2 | 2 | 2 | 4 | 2 | 3 | 2 |
| OVERALL CONDITION | | | 2 | 3 | 4 | 4 | 3 | 3 | 3 | 2 | 3 | 3 | 1 | 4 | 2 | 2 | 2 | 4 | 3 | 2 | 2 |

Please give details of any other problems or changes in your general health

Week 1: Very depressed and weepy.

Week 2: Sunday – chest pain.

Week 3: Thursday – woke several times during the night. Short of breath.

INSTRUCTIONS

Please complete this card every evening after your last meal, to tell us how you have been feeling during the past 24 hours; this will help us to make a thorough assessment of your illness and any treatment that you may need.

The codes to be used are shown on the reverse side of the week 5 section.

**Figure 1** Daily Diary Card.

chemotherapy (Maintenance – 'M') but at intervals of 4 weeks instead of 3.

*Physicians' assessment of quality of life*

Patients attended every 3 weeks during the induction period and every 4 weeks during the M/NoM period. At each attendance the clinician assessed the patient's overall condition and level of activity (Table I). In addition, the patient was asked about adverse reactions since the last attendance.

*Daily Diary Card*

At their first attendance upon entering the trial the patients were given the Daily Diary Card (Figure 1; Fayers & Jones, 1983), and were shown how to complete it every evening after their last meal, recording how they felt over the previous 24 h. Each card covered a period of up to 5 weeks, since although the clinic attendances were intended to be every 3 or 4 weeks, there were often delays. There were five questions, all graded on a five-point scale from one (most favourable) to five (least favourable); see Table I. Because the card was to be completed on a daily basis it was decided that only a few questions should be asked, and that they should have simple wording. As a consequence, although the patients were asked about 'mood' and 'anxiety' there was no attempt to use formally defined psychometric scales as would normally be implied by such terms. Although the questions about overall condition and physical activity are on a five-point scale for both the physicians' assessment and the diary card, there are minor differences in the wording used, particularly in the assessment of physical activity; this occurred because the physicians' assessment was in the same form as in previous MRC studies, whereas the diary card used simplified questions.

**Table I** Scales used on the Daily Diary Card and by the physician

| *Patients' assessment (Diary Card)* | *Physicians' assessment* |
|---|---|
| Overall Condition: | |
| 1. Very well | 1. Excellent |
| 2. Well | 2. Good |
| 3. Fair | 3. Fair |
| 4. Poor | 4. Poor |
| 5. Very ill | 5. Very poor |
| Physical Activity: | |
| 1. Normal work/housework | 1. At work or active retirement |
| 2. Normal work but with effort | 2. Full activity but not at work |
| 3. Reduced activity but not confined to home | 3. Out and about, but activity restricted |
| 4. Confined to home/hospital | 4. Confined to home or hospital |
| 5. Confined to bed | 5. Confined to bed |
| Vomiting: | |
| 1. None | |
| 2. Poor appetite | |
| 3. Felt sick but wasn't | |
| 4. Sick once | |
| 5. Sick more than once | |
| Mood: | |
| 1. Very happy | |
| 2. Happy | |
| 3. Average | |
| 4. Miserable | |
| 5. Very miserable | |
| Anxiety: | |
| 1. Very calm | |
| 2. Calm | |
| 3. Average | |
| 4. Anxious | |
| 5. Very anxious | |

### Results

*Patients in the study*

Between June 1981 and February 1985, 369 patients with limited disease were admitted to the study from 27 centres in the United Kingdom.

*Survival*

The median survival period from the start of treatment was 41 weeks (MRC Lung Cancer Working Party, 1989); of the 369 patients in the study, 196 (53%) who were alive and responding after the initial six courses of treatment were allocated at random to M (97 patients) or NoM (99). There was no statistically significant difference in survival between the M and NoM series ($P = 0.27$, log-rank test) although the median survival periods from randomisation were 37 weeks (M) and 29 weeks (NoM). One hundred and twenty-six patients were alive 6 months after randomisation, 54 at 1 year, 14 at 2 years and 11 at 3 years.

*Patients' compliance in use of diary card*

It was realised that a study collecting daily information about quality of life may have poorer compliance levels than if a questionnaire were used at the time of attending a clinic. Table II shows the level of compliance obtained with the Daily Diary Card. As few cards were received from patients in the last few weeks of life, the final month of survival was ignored whilst analysing the compliance. During the induction period 23% of the patients returned no information at all, and a further 28% returned cards covering less than half the induction period, or less than half their survival period, if they failed to survive that long. However there were marked differences between centres, with the patients from 37% of the 27 centres consistently returning most of their cards while at the other extreme those from one centre returned no information at all. Fortunately, from the statistical point of view, the level of compliance was similar in the M and NoM series, and thus between-treatment comparisons are likely to remain unbiased. However, the poor compliance raises the question as to whether the results are representative of what patients really feel about their treatment. We therefore carried out additional analyses: the baseline characteristics and survival were compared for patients who were 'good compliers' and 'bad compliers' (Table III, upper part). Similarly, quality of life assessments made by the physician at the time of entry to the study were compared for the two groups; the results are shown in Table III (lower part). No significant differences were found in age, but the patients returning most data appeared to be the healthier ones (Chi-squared for trend, $P < 0.02$ for physicians' assessments of activity and patients' self-assessment of overall conditions, $P < 0.001$ for physicians' assessments of overall condition).

**Table II** Compliance during the induction chemotherapy and during the first 3 months of follow-up period[a]

| *% of data received*[b] | *Induction* No. | *Induction* % | *Follow-up period M* No. | *Follow-up period M* % | *Follow-up period NoM* No. | *Follow-up period NoM* % |
|---|---|---|---|---|---|---|
| Nil | 80 | *23* | 34 | *36* | 43 | *44* |
| 1–50% | 95 | *28* | 13 | *14* | 18 | *18* |
| 51–100% | 167 | *49* | 47 | *50* | 37 | *38* |
| Total patients | 342 | *100* | 94 | *100* | 98 | *100* |
| Overall % of data returned in this period | 47 | | 48 | | 40 | |

[a]'Induction' is from the time of start until the date of allocation or, if no allocation was made, 155 days (the mean time until randomisation). If a patient died before allocation would have been due, date of death minus 1 month was used. 'Follow-up period' is the 3 months after allocation, or until date of death minus 1 month if this was earlier. [b]For example, a patient surviving 4 months and returning cards covering 1 month would be classified under '1–50% of data received'.

**Table III** Percentage of Diary Card data returned, according to patients' age, survival and clinical condition

| | *Percentage of Diary Card data returned* 0% | 1–50% | 51–75% | 76–100% |
|---|---|---|---|---|
| Number of patients | 74 | 83 | 60 | 78 |
| Median age (years) | 59 | 59 | 59 | 58 |
| Median survival (days) | 343 | 348 | 338 | 358 |
| (no significant survival differences: log-rank test) | | | | |
| *Percentage of patients with* | | | | |
| Physicians' assessment of pretreatment level of physical activity: Not restricted (grade 1 or 2) | *55* | *59* | *68* | *71* |
| Physicians' assessment of pretreatment overall condition: Excellent/good (grade 1 or 2) | *63* | *61* | *73* | *85* |
| Patients' assessment of overall condition until time of second course: Good or excellent (75% of time at grade 1 or 2) | – | *79* | *89* | *93* |
| Physicians' assessment of overall condition at time of second course: Good or excellent (grade 1 or 2) | *55* | *59* | *77* | *81* |

### Quality of life

### *Quality of life charts*

Figure 2 shows the percentage of patients reporting themselves in the worst two grades, namely 4 to 5 (see Table I), for each day in the first year from the start of induction chemotherapy. The charts for activity were similar to those for other questions and to conserve space are not displayed. The figure is confined to those patients alive at 6 months. The number of patients surviving decreased substantially as the study progressed; during the first course 116 patients (52 M and 64 NoM) completed cards, but by 40 weeks the number of patients returning information was 61 (37 M and 24 NoM).

Although the chemotherapy (Ct) was scheduled to be given at intervals of 3 weeks (induction) or 4 weeks (maintenance), in practice it was sometimes delayed for reasons such as toxicity or holidays; however, the total amount of delay was similar in the M and NoM groups. Since each course of chemotherapy is likely greatly to affect the general health of the patients, the mean time between each course was calculated and the results for each patient were realigned to this schedule. The figure clearly shows the effect of chemotherapy in producing a few days of diminished quality of life. The pattern of cycles is broken by the radiotherapy (Rt), which was given after the first two courses. The M group continued to display the same pattern throughout the 12 courses of chemotherapy, but there were clear signs of deterioration in the NoM group about 15 weeks after their last course, with higher proportions of patients reporting themselves in grades 4 to 5. The NoM patients showed a slight increase in vomiting during the later weeks: this may be explained by some receiving extra palliative treatment, mainly cyclophosphamide.

One particularly interesting observation is that most of the charts in Figure 2 also show a small deterioration in quality of life half way between successive courses. We believe that this may be associated with the nadir in blood count usually observed at this time. It may also be noted that there is a prolonged deterioration after the course of chemotherapy that follows the radiotherapy, reflecting the combined haematological toxicity of chemotherapy and radiotherapy; we are unable to explain why this effect is more marked in one group than the other, despite their receiving identical treatment.

Taking the charts pair by pair, there are suggestions that
(a) Overall condition: the no maintenance group has a slightly smaller percentage of patients in grades 4 or 5 during treatment, despite their receiving identical treatments; however they deteriorated more after week 40 (although the maintenance group continued to show treatment-related peaks).
(b) Vomiting: the maintenance group continued to show peaks associated with continuing treatment.
(c) Mood: The no maintenance group was worse after week 28, except for small peaks during maintenance treatment.
(d) Anxiety: The pattern was the same as for mood.

### *Validity*

Table IV compares the overall condition on the Daily Diary Card with the results of the physicians' assessment at the time of a course of chemotherapy. Since the patients had recorded daily information the scores were averaged. The table shows simple arithmetic means calculated by coding the categories 1 to 5; the scores were calculated within four groups. For this comparison the unit of observation was the course, not the patient, and so each patient may have been

**Figure 2** Percentage of patients reporting themselves in categories 4 or 5, plotted against the number of weeks since start of treatment.

counted several times. Forty-five per cent of assessments were, using this scaling, identical and 39% were worse when made by the patient rather than the physician. However it should be noted that there is little reason to equate the patients' scoring with the physicians' assessments using five groups with the arbitrary boundaries that we chose to use, and very different results could easily be obtained by choosing a different scale for the comparison. We also examined the data by weighting the scores recorded close to the time of the clinic attendance more heavily than earlier scores, so that the mean is a better reflection of the patients' condition at the time of the physician's assessment. This made little difference to the results presented here.

Table V compares nausea and vomiting since the last course of treatment, as reported by the patient and by the physician; the patients reported more vomiting on the Diary Card (vomiting during 626 courses) than was reported by the physician (360 courses). Additional comparisons, not presented, showed close agreement with respect to patients' and physicians' assessment of activity. Also, poor overall condition was associated with weight loss since the previous visit; Table VI shows, for example, that of the patients graded 4, 51 (58%) lost weight, 27 (31%) did not change, 10 (11%) gained weight. The corresponding results for patients graded 1 were 25 (23%), 43 (39%) and 41 (38%).

### *Differences between courses*

Table VII shows the duration of vomiting following each course. As patients continued to receive successive courses there was no significant change in the number of days in which they vomited. Over the six courses, for example,

**Table IV** Comparison of overall condition, as assesssed by physician and by patient: number of patients at each grade

| *Physicians' assessment* | *Patients' assessment[a]* 1 | 2 | 3 | 4 | *Total* |
|---|---|---|---|---|---|
| 1. Excellent | 61 | 97 | 43 | 3 | 204 |
| 2. Good | 69 | 315 | 353 | 20 | 757 |
| 3. Fair | 20 | 90 | 247 | 47 | 404 |
| 4 + 5. Poor | 0 | 6 | 50 | 26 | 82 |
| Total | 150 | 508 | 693 | 96 | 1447 |

[a]Patients' assessment = average over previous 3 weeks, grouped as: 1 = less than 1.5; 2 = 1.5 to 2.49; 3 = 2.5 to 3.49; 4 = 3.5 or more.

**Table V** Comparison of patients' and physicians' assessments of nausea and vomiting following the first six courses of chemotherapy: number of courses reported

| *Physicians' assessment* | *Patients' assessment* No data | None | Nausea | Vomiting | Total |
|---|---|---|---|---|---|
| Not reported | 554 | 125 | 78 | 371 | 1128 |
| Nausea | 80 | 27 | 31 | 79 | 217 |
| Vomiting | 115 | 49 | 20 | 176 | 360 |
| Total | 749 | 201 | 129 | 626 | 1705 |

**Table VI** Patients' assessment of overall condition compared with weight loss over the sample period

| *Weight change* | *Patients' assessment[a]* 1 | 2 | 3 | 4 | *Total* |
|---|---|---|---|---|---|
| Weight loss | 25 | 95 | 210 | 51 | 381 |
| No change (less than 1 kg) | 43 | 179 | 228 | 27 | 477 |
| Weight gain | 41 | 132 | 140 | 10 | 323 |
| Total | 109 | 406 | 578 | 88 | 1181 |

[a]Same scale as Table IV.

**Table VII** Percentage of patients according to days of vomiting following each course[a]

| *Days* | *Course* 1 | 2 | 3 | 4 | 5 | 6 |
|---|---|---|---|---|---|---|
| 0 | 27 | 22 | 16 | 28 | 25 | 19 |
| 1 | 37 | 36 | 42 | 28 | 27 | 21 |
| 2 | 20 | 25 | 24 | 24 | 31 | 44 |
| 3 | 5 | 10 | 4 | 9 | 12 | 11 |
| 4 | 3 | 2 | 3 | 2 | 1 | 2 |
| 5+ | 8 | 4 | 11 | 8 | 4 | 3 |
| Number of patients | 60 | 130 | 100 | 96 | 84 | 63 |

[a]At each course data were only included if the patient had provided data for all of the following 7 days.

similar percentages of patients reported 2 or more days of vomiting. Similarly, no differences between courses were seen in the answers to the other questions on the diary card.

*Anxiety preceding a course of treatment*

Although there was little suggestion from the charts, it was thought possible that patients might become anxious or miserable in the few days prior to a course of treatment. We therefore analysed in more detail the numbers of patients reporting themselves to be anxious during the 7 days prior to the 5th course during the induction period. The fifth course was chosen because by this time the patients are familiar with the effects of the treatment; the sixth course might not be representative, as some of the NoM patients would have already been told that this is their last course. No patterns were evident in the numbers, although it is perhaps likely that a few patients may become more anxious immediately prior to visiting the hospital whilst others may feel relieved to be going back for treatment. Additional analyses of the data preceding other courses showed similar lack of patterns.

*Comparison of M and NoM groups*

In addition to the graphical representations, it is possible to summarise the data in various numerical ways. Table VIII examines the percentage of days that patients rated themselves in each category during the 6-week period after the end of induction treatment. More M than NoM patient-days were reported in the worse categories (3 to 5) for all questions, which agrees with the quality of life charts in which, after the induction period, the area under the curves is much larger for M patients than for NoM patients.

Table IX, using a different style of presentation, gives the number of days that patients graded themselves as having poor overall condition (categories 4, 5). By the second period larger numbers of the M patients felt ill than NoM patients, although initially eight of the 22 NoM patients assessed were in categories 4/5 for at least 5 days.

### Discussion

*Analysis of Diary Cards*

The diary card has produced large amounts of information with the 369 patients returning on average more than 100 days of repeated data, representing a total of nearly 200,000 items of information. A natural method of analysis to consider is to reduce the data for each patient to a few summary scores, such as 'average overall condition'. We have used this approach in some of the tables. However, there are obvious difficulties in using any form of averaging process, especially when attempting to combine severity with duration. For example, is 1 day of feeling very ill followed by a day of feeling very well equivalent to 2 'average' days? Furthermore, if one wishes to use average scores surely it is preferable, not to say far simpler to implement, to ask the patient to carry out the averaging and weighting by posing such questions as 'How have you been feeling since your last course of treatment?'.

**Table VIII** Percentage of patient days in each category in the 6 weeks following course 6[a]

| *Category* | *Overall condition* M | NoM | *Physical activity* M | NoM | *Vomiting* M | NoM | *Mood* M | NoM | *Anxiety* M | NoM |
|---|---|---|---|---|---|---|---|---|---|---|
| 1 | 12 | 14 | 21 | 31 | 75 | 84 | 7 | 8 | 7 | 12 |
| 2 | 28 | 39 | 16 | 24 | 9 | 7 | 28 | 33 | 33 | 34 |
| 3 | 49 | 38 | 42 | 36 | 7 | 5 | 52 | 48 | 48 | 41 |
| 4 | 10 | 8 | 18 | 8 | 2 | 1 | 11 | 10 | 9 | 11 |
| 5 | 1 | 1 | 3 | 1 | 6 | 3 | 2 | 1 | 3 | 1 |

[a]Based on 47 M and 37 NoM patients with at least 50% of data available.

**Table IX** Days at overall condition of poor or very ill (categories 4, 5), following course 6[a]

| *Days* | *0–4 weeks* M | NoM | *5–8 weeks* M | NoM |
|---|---|---|---|---|
| 0 | 15 | 8 | 16 | 20 |
| 1 | 4 | 3 | 0 | 1 |
| 2 | 5 | 2 | 1 | 0 |
| 3 | 0 | 1 | 3 | 0 |
| 4 | 1 | 0 | 0 | 1 |
| 5+ | 1 | 8 | 5 | 1 |
| Patients | 26 | 22 | 25 | 23 |

[a]Only includes patients with all relevant data.

The primary objective of using a diary card was to collect detailed information, so as to explore the patterns of changing quality of life during the cycles of treatment. This does, however, result in large quantities of data which are difficult to summarise and interpret. The ratings for each question are on discrete five-point ordinal scales which do not necessarily have linear intervals; there is much missing data with patients failing to complete data for individual days, not returning one or more cards, or completely ceasing to fill in cards; since many patients died within the first few months, there is 'censoring' of the data which may possibly be related to deteriorating quality of life; patients may have courses of treatment delayed, omitted, or the dosage reduced, either because of toxicity and impaired quality of life or simply because the patient goes on holiday. Difficulties of analyses and interpretation bedevil the use of any diary cards, and workers in other fields have experienced similar problems with daily data (Machin *et al*., 1987). We have therefore attempted to analyse and present the results in a variety of tables and formats, but the simplest and most useful display is perhaps that of the Figure 2. Whilst not being amenable to formal statistical significance testing, these charts quite vividly show the changing quality of life between and across courses of treatment, and appear adequately to reflect the differences between the policies of treatment. Although we have displayed separate charts for each question on the card, the patterns are all closely similar and use of a Likert or summated scale might be considered as an alternative to this battery of charts. However, it is doubtful whether a simple summated scale would be able to represent the overall quality of life, and other studies have suggested that it may be preferable to report separate scores (Selby *et al*., 1984; Stewart *et al*., 1981).

### *Compliance*

Compliance is often a problem with self administered assessments (Baum *et al*., 1979), and depends crucially upon the manner in which the patient is introduced to the questionnaire (van Dam *et al*., 1983). Such problems are likely to be greater both for a multicentre study as opposed to a single centre study, and for a daily diary card than for a questionnaire completed when the patient attends for treatment. However, reports from many centres suggested that if time was taken to explain that the card has two benefits, namely in assisting with the review of the patients' condition and in helping to investigate the problems of the treatment, most patients were willing to complete cards regularly. This was confirmed by the way that those physicians who most successfully used the card, and who presumably also most encouraged their patients to make full use of it, found that patients made much use of the blank space at the bottom of the card to describe how they felt during their treatment. We observed major differences in the level of patient compliance between centres, again suggesting that much of the problem relates to the way in which the cards were administered and confirming the need to ensure that efforts were made to provide sufficient motivation for the patients. Those centres with additional support staff assigned to clinical trials were likely to obtain higher rates of compliance than other centres. However, the overall levels of compliance were the same in both the M and NoM group, and we therefore believe that bias is unlikely to render tests for differences between the two groups of patients invalid. Patients returning few or none of the cards may differ from patients returning larger numbers; although age and survival did not appear to relate to compliance there did appear to be an association of compliance with the patient's overall condition. In particular, there was a suggestion that healthier patients were more likely to complete cards. However, this effect was present in both groups and is therefore unlikely to bias the results. Thus, notwithstanding the variable level of compliance, we consider the quality of life data collected during this trial to be representative of the patients entered into the study.

### *Validity*

It is generally recognised that validity is of crucial importance in assessing any new scale (e.g. Feinstein *et al*., 1986; Goldberg & Hillier, 1979; Kaplan *et al*., 1976; Nunnally, 1978). Unfortunately, however, there is a growing awareness that for quality of life measurements there is no 'gold standard' against which to compare scales (Boyd *et al*., 1988; Derogatis & Spencer, 1984; Kaplan *et al*., 1976; Selby *et al*., 1984; Till *et al*., 1984) and that indirect methods must be used. In theory an in-depth psychological interview might seem to be the ideal yardstick, but in the context of a multicentre trial this is usually impractical. More importantly, however, the act of conducting an in-depth interview is very likely to modify a patient's perception of their quality of life (Brinkley, 1985); many patients are relieved to be able to discuss how they feel (van Dam *et al*., 1983; Fallowfield *et al*., 1987; Clark & Fallowfield, 1986), and appreciate the interest that is shown in their quality of life.

Instead one can consider comparing the new scale against existing instruments. In this case, however, the current authors would argue that if two scales ask broadly similar questions about, say, overall condition then it would be most surprising if the results obtained from both were not also broadly similar; on the other hand it would be surprising if the results were identical. Furthermore, if the results *were* identical it would merely demonstrate that the new scale confers no advantages. Thus in general it is of limited interest to compare two different scales, unless the newer one has advantages such as being simpler to administer or providing more useful information. Also, the degree of agreement between self-assessment of health and physician ratings has been found to vary from very litle to very high according to the topic being assessed and it has been suggested that it may be most practical to choose a scale which is not necessarily 'true' in some absolute sense, but rather which is most useful in providing comparative data (Hunt *et al*., 1980).

Undaunted, however, we compared the results obtained from the clinicians assessment with those from the Daily Diary Card. In order to make the comparisons it was necessary to average the patients' daily scores, which implicitly makes the dubious assumptions that the scales are linear (so that 1 day at grade 2 followed by 1 day at grade 4 is equivalent to 2 days at grade 3), and also that the physicians' assessments represent averages of how the patients have felt since the previous assessment. Although the questions differed slightly in wording and thus were not directly comparable, we nevertheless found reasonable agreement. One particular aspect was of special interest: the patients reported much higher levels of vomiting after their courses of chemotherapy than had been recorded by the physicians. However, the form completed by the physicians did not contain an explicit question about vomiting, but merely a general heading of 'adverse reactions'. Since it is likely that the chemotherapy would induce vomiting in most patients, there seems to be evidence that the physicians were under-reporting the incidence of vomiting whilst the self-assessment with the diary card is likely to be a more accurate representation of what was happening. There was close agreement between the patient's and physician's assessment of activity. Also, there was strong association between the patients' assessments of overall condition and weight loss since the previous visit.

The patients also answered questions about 'mood' and 'anxiety'; these were loosely worded questions and it is in no way claimed that they provide a formal assessment of the analogous psychometric terms; however, we expect the questions to provide information about the patients' perception of their general condition. These scores showed similar associations and patterns as the other measures, and appeared to contribute little additional information. The clear patterns in the charts and their interpretation as described in the results strongly suggest that the diary card is yielding valid data, some of which are not available from less frequent assessments. This claim is also supported by the results in Geddes *et al*. (1990), who compared the diary card, in a version very similar to ours, against EORTC and Spitzer quality of life assessments; they concluded that the card succeeded in measuring the more specific variables of the EORTC questionnaire, but did not address all the areas of the Spitzer index.

*Sensitivity and reliability*

The charts show that the diary card was sensitive enough to detect adverse effects of treatment, particularly the large day-to-day changes in general health which occur following a course of chemotherapy. The period of radiotherapy is also apparent, as is the deterioration in health in NoM patients after the end of treatment. The time when blood counts are usually at their nadir is also visible; this also corresponds to the uneven distribution of deaths during chemotherapy, noted elsewhere (MRC Lung Cancer Working Party, 1989; Souhami *et al.*, 1987), which occurs most often about 10 days after a course. Similarly, the consistency of the effects on successive courses and in both the M and NoM groups supports the proposition that the card yields repeatable, sensitive and reliable data. Geddes *et al.* (1990) found that the diary card gave reproducible data which reflected the day to day variation in symptoms during chemotherapy in a way which the other questionnaires could not.

In summary, it would appear that the diary card is sufficiently sensitive to detect time and treatment related changes, and is also sufficiently reliable to be able to yield repeatable measurements. The results in the charts, with their natural interpretation, also confirm that the Daily Diary Card appears to be a valid measure of general health.

*Consequences of poor compliance*

As commented above, compliance has frequently been found to be a problem in quality of life assessments. What has less often been noted is that lack of compliance in quality of life reporting may lead to serious biases the magnitudes of which are difficult to assess. For example, it may be that patients with poor quality of life will more often refuse to answer or, alternatively, it may be those with favourable quality of life who see little point in reporting how they feel. The former appeared to be the case in our study, and better compliance was obtained in healthier patients. Although the between-group comparisons in the present study are likely to remain valid, especially since the compliance levels were similar in the two groups, it is difficult to know how to interpret the results in terms of the overall health of patients. It should also be borne in mind that patients in a clinical trial may not be representative of patients treated under routine conditions. In particular, the patients may have been more closely monitored and supervised, and the very act of completing and returning Diary Cards may have affected the patients' perception of the care and support that was being given. Even with a high level of compliance, it would remain unclear as to how applicable the results obained from a scientific investigation are as an indicator of the quality of life to be expected in future patients undergoing the same policy of treatment. Whilst this is also true for most outcomes from clinical trials in general, it is possible that subjective assessments such as Quality of Life are more strongly influenced.

*Quality of life in clinical trials*

Patients in clinical trials are often followed-up more closely than those treated in routine practice, and the quality of their treatment may also be better: this frequently leads to differences even in objective measurements such as length of survival, with more favourable results being seen in patients participating in a trial. It is even more likely that quality of life experienced by patients in a clinical trial will differ from that of patients outside a trial, and even the act of completing a daily card may influence their perception of their condition. It is difficult to know to what extent the absolute values of the measurements from clinical trials will apply in general clinical practice, although any differences detected between study treatment groups are likely to remain valid. It is also important to consider how quality of life assessments obtained in a trial may best be used subsequently to affect the decision process faced by clinicians when deciding how to allocate or modify the treatment of their patients, since the experiences of those patients may prove to be very different from those of the trial patients. Some attempts have been made to weigh quality of life against duration of survival (McNeil *et al.*, 1981), also in the form of Quality-adjusted life years (QALYs) (Williams, 1985; Kind *et al.*, 1982), but such approaches remain controversial (Smith, 1987).

### Conclusions

In this study there was no clear evidence that extending chemotherapy beyond six courses prolonged survival, although there was a suggestion that it may be beneficial in patients showing a complete response to initial chemotherapy. However, the results from the Diary Card show that most of the adverse side effects appear to be confined to the first few days following a course of chemotherapy, although there is also a small deterioration which may be associated with the 'nadir' effect. This applied to all the dimensions assessed, namely overall condition, physical activity, vomiting, mood and anxiety. These results should assist physicians in discussing the likely effects of treatment with patients, and in counselling them. However, over 50% of the patients returned less than half of their cards and there appeared to be a slight tendency for better compliance in the healthier patients, possibly suggesting that side effects may have been underestimated. We have also shown that some patients allocated to No Maintenance experienced a gradually deteriorating quality of life, as opposed to the brief but more severe side effects which continued to occur following each course in the Maintenance group. This supports the hypothesis that there was a palliative effect in the M group. We believe the Daily Diary Card has enabled the effects of the treatment to be examined in more detail than by using more conventional methods, and the Medical Research Council Lung Cancer Working Party is continuing to use similar cards in subsequent studies.

The following consultants and their colleagues participated in the study: Amersham: A.O. Robson; Bangor: N.G. Hodges; Basingstoke: J.M. Fowler; Belfast: W.P. Abram; J.I. Coyle, W. Craig Martin, J. MacMahon, D.R.T. Shepherd, G. Varghese; Bradford: A.J. King, D.A.G. Newton; Brighton: H.I. Bijapur, J.P.R. Hartley, N. Hodson, C.W. Turton; Bristol: S. Goodman, E.C. Whipp; Cambridge: N.M. Bleehen; Clatterbridge: M.J. Garrett, D.C. Hurman, A.J. Slater; Glasgow: G.W. Allan, I. McHattie, A.R. Russell, R.P. Symonds, H.M.A. Yosef; Hammersmith: K.E. Halnan, C.G. McKenzie; Hexham: R.G. Brackenridge, J.B. Ryder; High Wycombe: W.B. Thomson; Ipswich: C.R. Wiltshire; Lanarkshire: J.C.J.L. Bath; Leeds: D.V. Ash, H.J. Close, M.F. Muers, J. Stone; Margate: R.H. Andrews; Middlesborough: H.R. Gribbin, N.L.K. Robson, P. Ryan; Middlesex: M. Spittle; Mount Vernon: S. Dische, M.I. Saunders; Newcastle: J.M. Bozzino, G.J. Gibson, D.J. Hendrick, J. Lauckner, S. Nariman; Oxford: M.K. Benson, J.M. Hopkin, A.H. Laing, D.J. Lane, R. Marshall; Swindon: J.A. Waddell; Southampton: D.J. Lipscomb, R.D.H. Ryall; Wolverhampton: D.J. Fairlamb; York: A.M. Hunter. Local coordinators were D. Barron, J. Boyle, R. Collins, C. des Rochers, D. Evans, A. Fenwick, S. Jayne, K. McGregor, S. Morrow, S. Mucur, A. Pickett, J. Pye, D. Robinson, G. Sainsbury, M. Stewart, S. Ward and T. Young. The reference histopathologist was Dr P.G.I. Stovin. We are grateful to Bristol Myers, Slough, for their assistance with supplies of etoposide.

### References

BAUM, M., PRIESTMAN, T. & JONES, E.M. (1979). A comparison of quality of life in a controlled trial comparing endocrine with cytotoxic therapy for advanced breast cancer: In Mauridson, H.T. & Palshof, P. (eds) *Breast Cancer: Experimental and Clinical Aspects*. Pergamon Press: London.

BOYD, N.F., SELBY, P.J., SUTHERLAND, H.J. & HOGG, S. (1988). Measurement of the clinical status of patients with breast cancer: evidence for the validity of self assessment with linear analogue scales. *J. Clin. Epidemiol.*, **41**, 243.

BRINKLEY, D. (1985). Quality of life in cancer trials (editorial). *Br. Med J.*, **291**, 685.

CLARK, A., FALLOWFIELD, L.J. (1986). Quality of life measurements in patients with malignant disease: a review. *J.R. Soc. Med.*, **79**, 165.

DEROGATIS, L.R. & SPENCER, B.S. (1984). Psychometric issues in the psychological assessment of the cancer patient. *Cancer*, **53**, 2228.

FALLOWFIELD, L.J., BAUM, M. & MAGUIRE, P. (1987). Do psychological studies upset patients? *J.R. Soc. Med.*, **80**, 59.

FAYERS, P.M. & JONES, D.R. (1983). Measuring and analysing quality of life in cancer clinical trials: a review. *Stat. Med.*, **2**, 429.

FEINSTEIN, A.R., JOSEPHY, B.R. & WELLS, C.K. (1986). Scientific and clinical problems in indexes of functional disability. *Ann. Intern. Med.*, **105**, 413.

GEDDES, D.M., DONES, L., HILL, E. & 5 others (1990). Quality of life during chemotherapy for small cell lung cancer: assessment and use of a daily diary card in a randomised trial. *Eur. J. Cancer*, **26**, 484.

GOLDBERG, D.P. & HILLIER, V.F. (1979). A scaled version of the General Health Questionnaire. *Psychol. Med.*, **9**, 139.

HUNT, S.M., McKENNA, S.P., McEWEN, J., BACKETT, E.M., WILLIAMS, J. & PAPPE, E. (1980). A quantitative approach to perceived health status: a validation study. *J. Epidemiol. Comm. Hlth.*, **34**, 281.

KAPLAN, R.M., BUSH, J.W. & BERRY, C.C. (1976). Health status: types of validity and the Index of Well Being. *Health Services Res.*, **11**, 478.

KIND, P., ROSSER, R., WILLIAMS, A. (1982). Valuation of quality of life, some psychometric evidence. In Jones-Lee, M.W. (ed.) *The Value of Life and Safety*. North Holland Publishing Company: Amsterdam.

MACHIN, D. D'ARCANGUES, C., BUSCA, B., FARLEY, T.M.M. & PINOL, A. (1987). Vaginal bleeding patterns – the problem and an example data set. *Appl. Stochastic Models and Data Analysis*, **3**, 27.

MEDICAL RESEARCH COUNCIL LUNG CANCER WORKING PARTY (1989). Controlled trial of 12 versus 6 courses of chemotherapy in the treatment of small-cell lung cancer. *Br. J. Cancer*, **59**, 584.

McNEIL, B.J., WEICHSELBAUM, R. & PANKER, S.G. (1981). Tradeoffs between quality and quantity of life in laryngeal cancer. *N. Engl. J. Med.*, **305**, 982.

NUNNALLY, J.C. (1978). *Psychometric Theory*. 2nd Edn. McGraw-Hill: New York.

SELBY, P.J., CHAPMAN, J.A.W., ETAZADI-AMOLI, J., DALLEY, D. & BOYD, N.F. (1984). The development of a method for assessing the quality of life of cancer patients. *Br. J. Cancer*, **50**, 13.

SMITH, A. (1987). Qualms about QALYs. *Lancet*, **i**, 1134.

SOUHAMI, R.L., MORITTU, L., EARL, H.M., ASH, C.M. (1987). Identification of patients at high risk of chemotherapy-induced toxicity. *Proceedings of IASLC Workshop on Combined Modality Therapy in Lung Cancer*: Le Havre.

STEWART, A.L., WARD, J.E., BROOK, R.H. (1981). Advances in the measurement of functional status: construction of aggregate indexes. *Med. Care*, **19**, 473.

TILL, J.E., McNEIL, B.J., BUSH, R.S. (1984). Measurement of multiple components of quality of life. *Cancer Treatment Symposia*, **1**, 177.

VAN DAM, F.S.A.M., LINNSEN, A.C.G., COUZIJN, A.L. (1983). Evaluating quality of life: behavioural measures in clinical cancer trials. In Staquet, M., Sylvester, R., Buyse, M. (eds) *The Practice of Clinical Trials*: Oxford University Press.

WILLIAMS, A. (1985). Economics of coronary artery bypass grafting. *Br. Med. J.*, **291**, 326.

WORLD HEALTH ORGANIZATION (1979). *WHO Handbook for Reporting Results of Cancer Treatment*. WHO Offset Publication No. 48. WHO: Geneva.


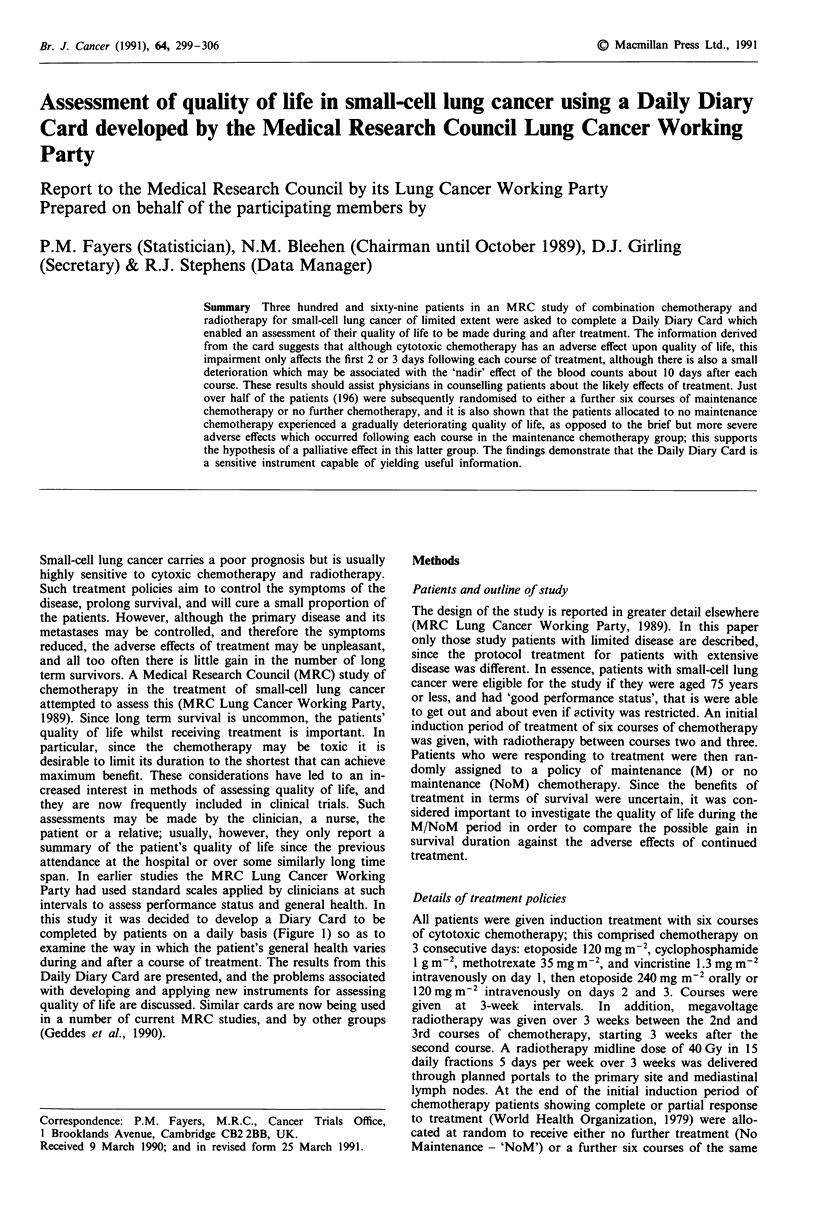

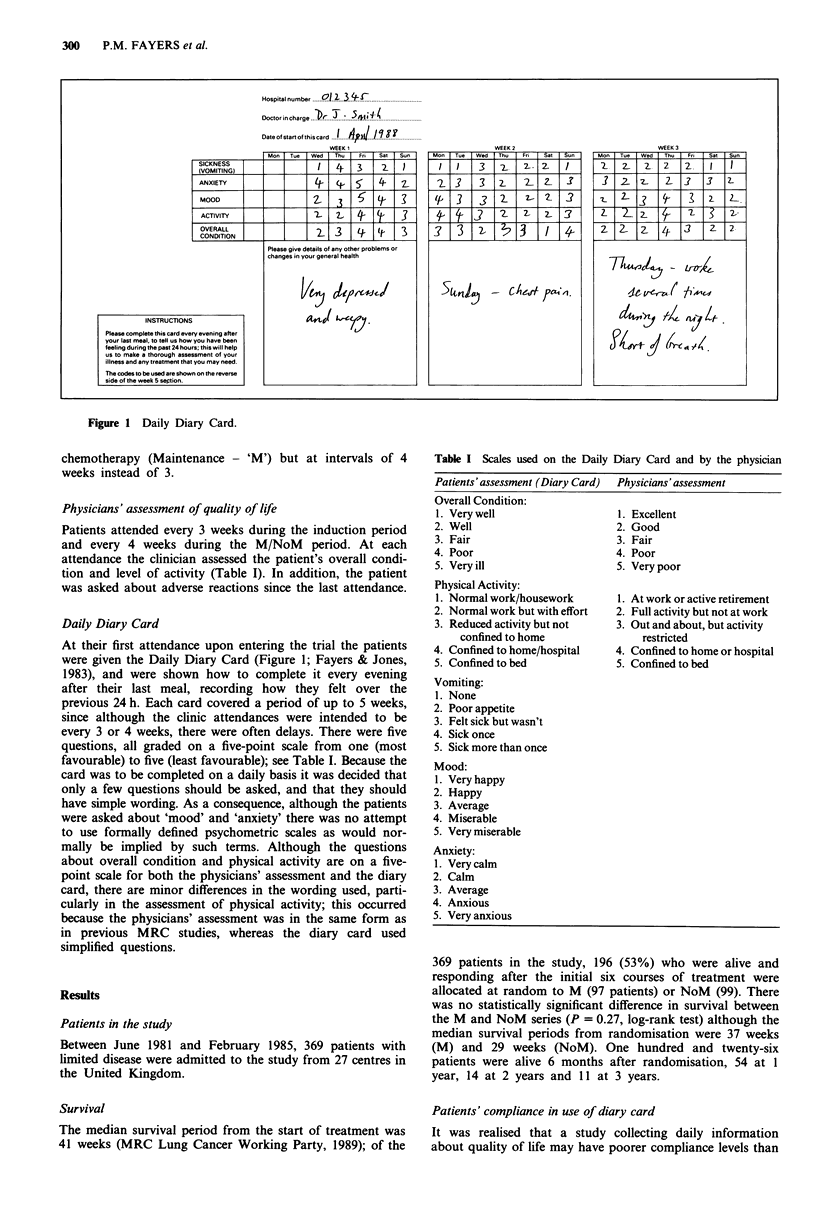

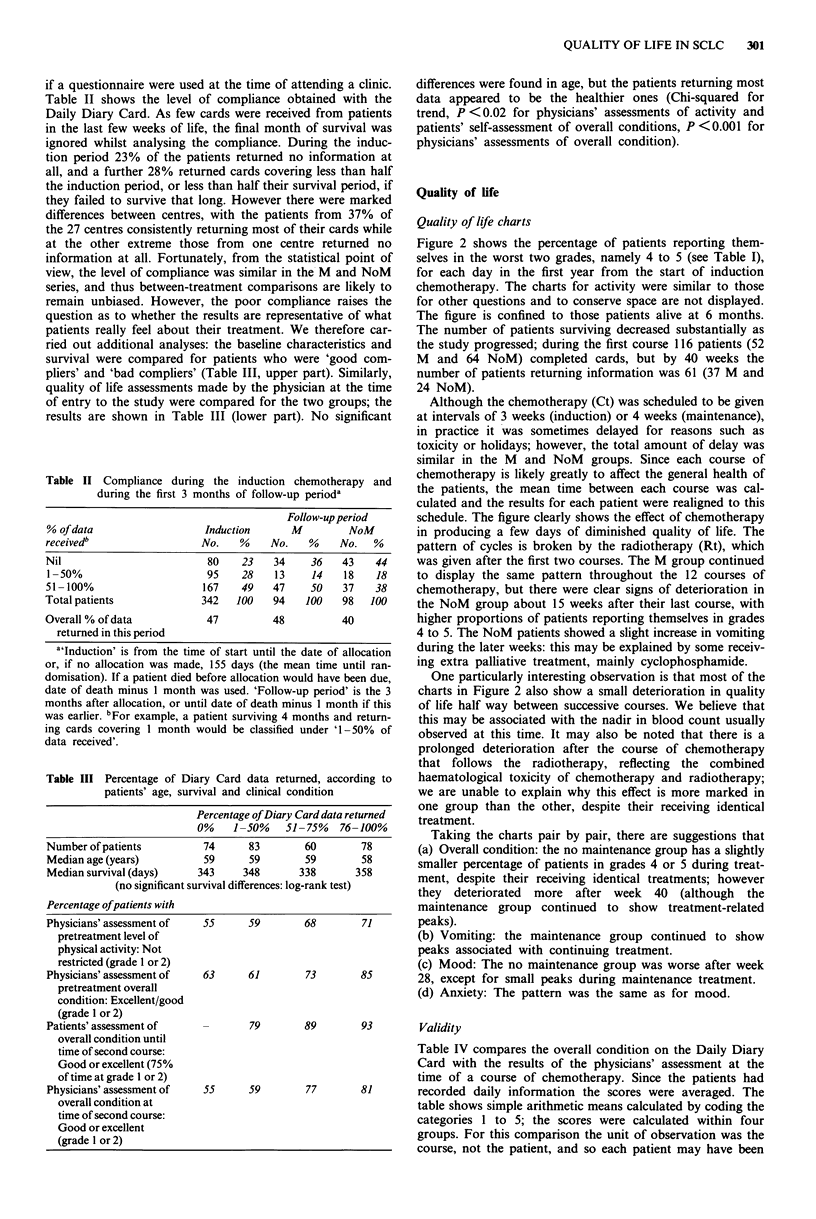

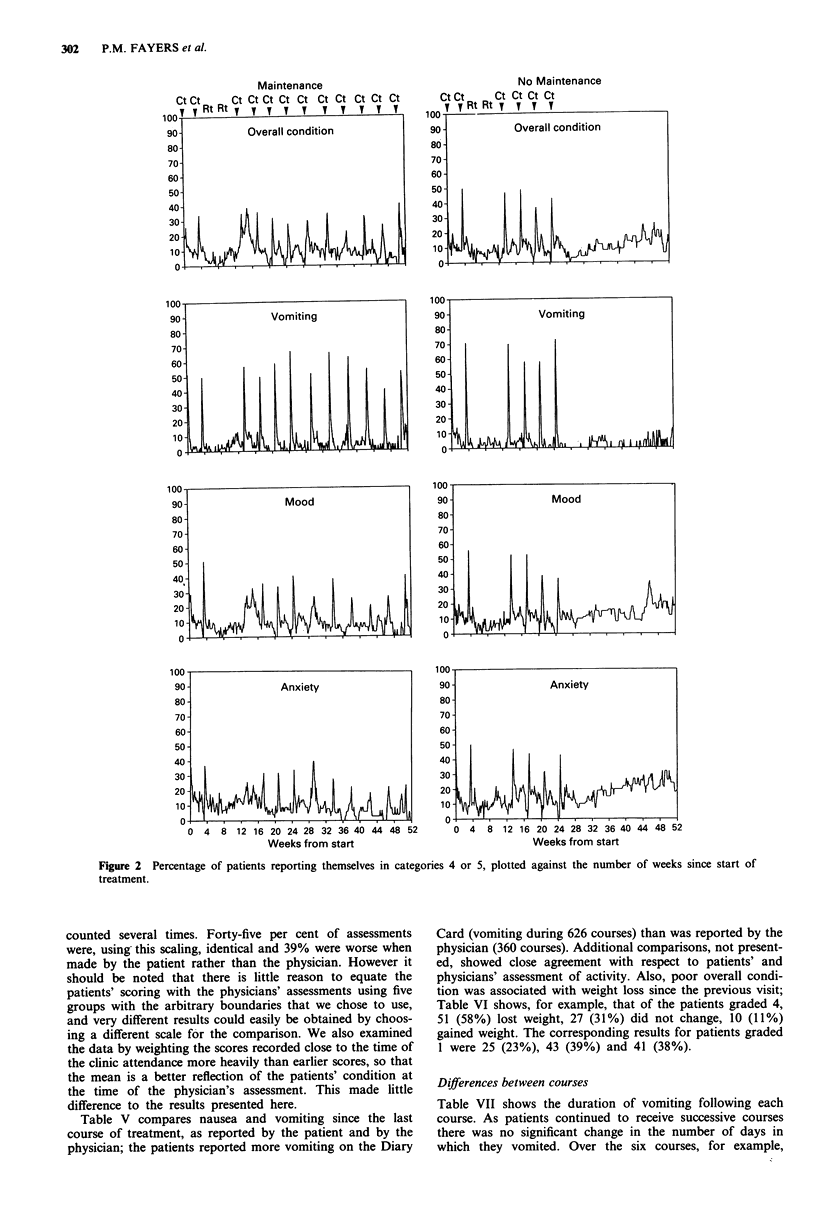

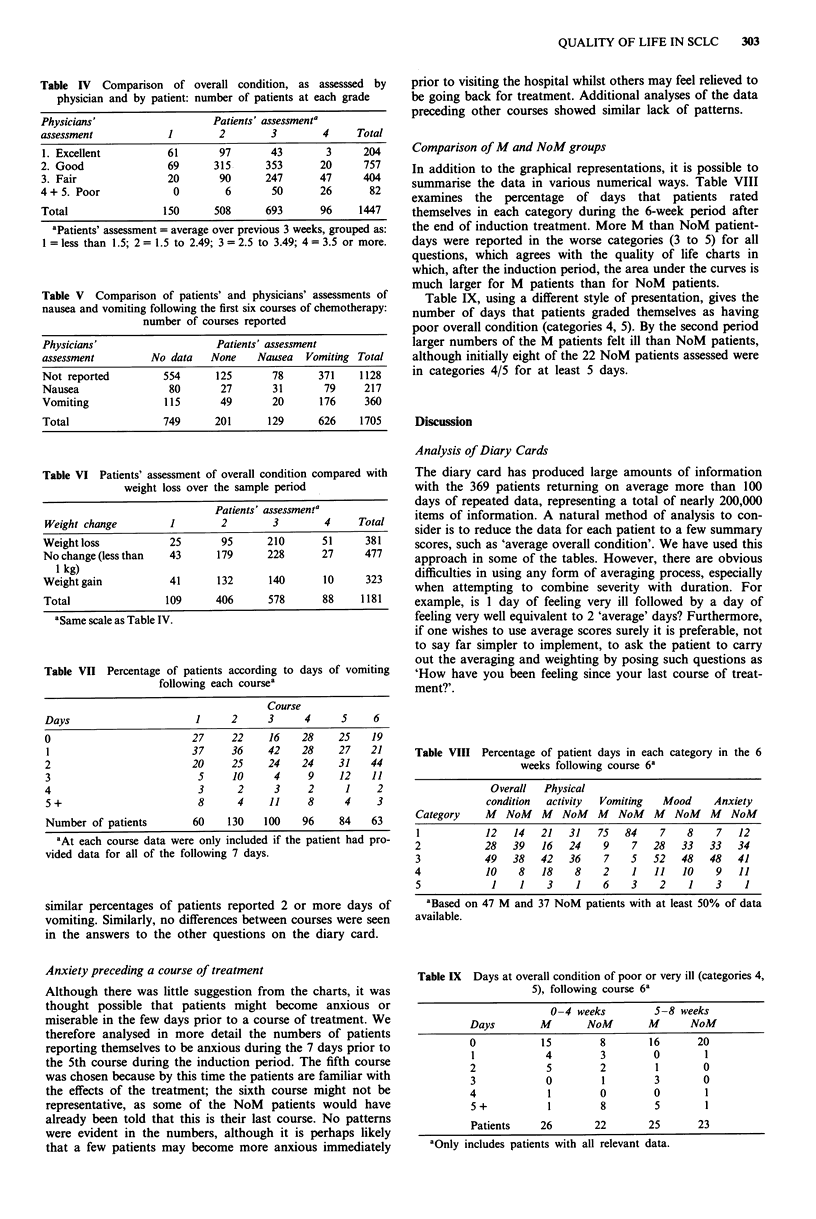

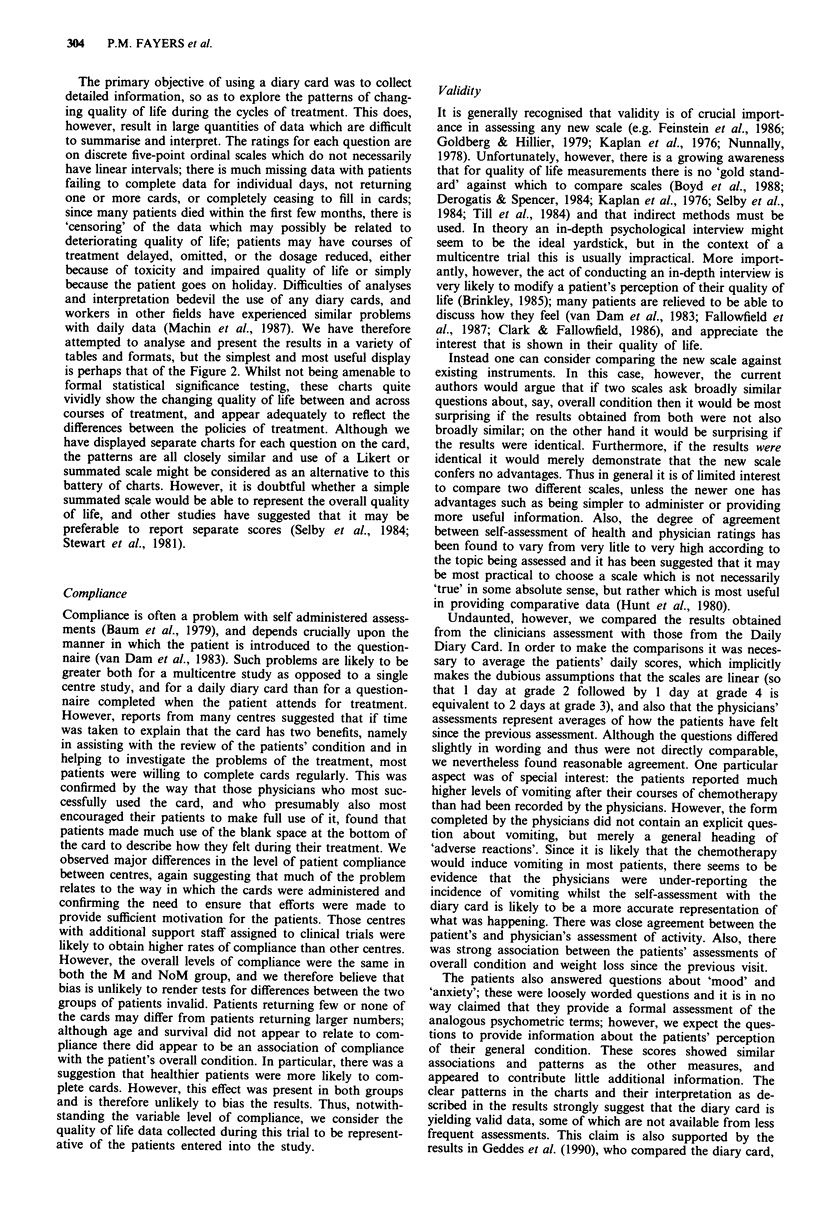

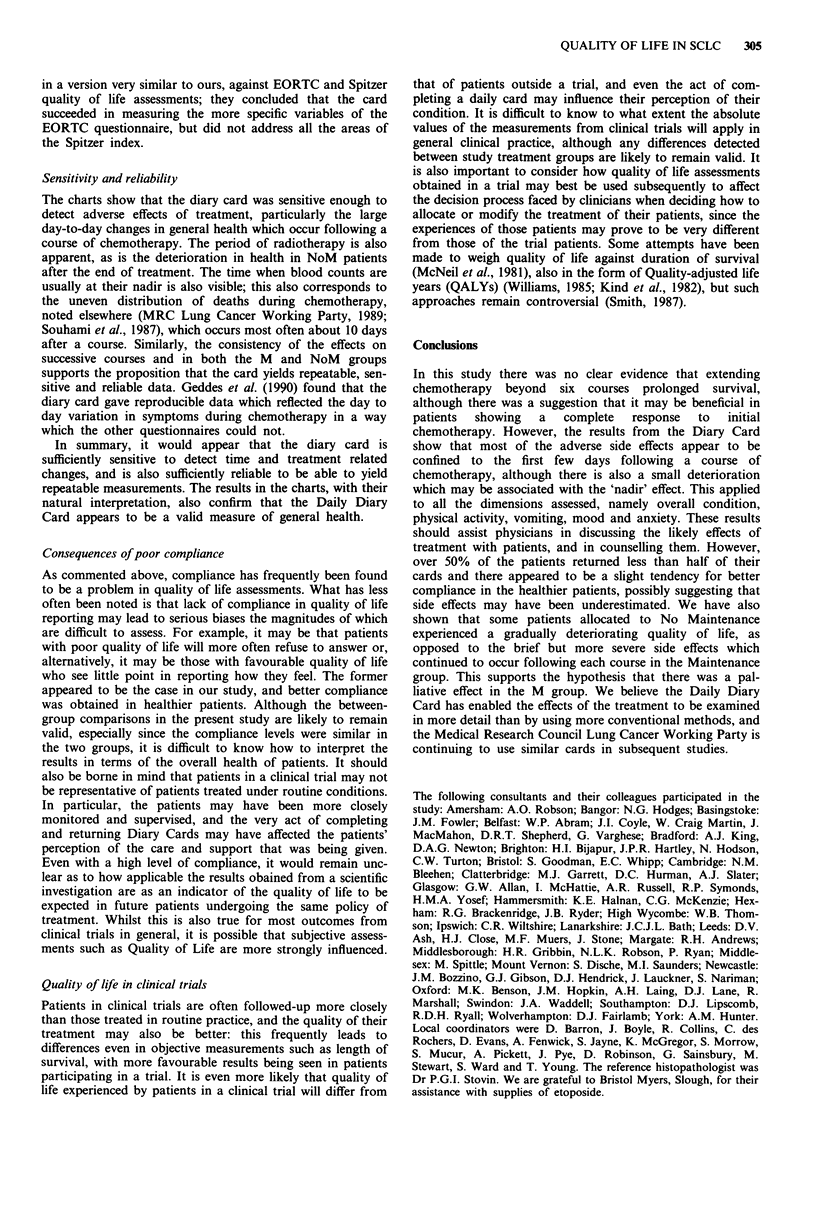

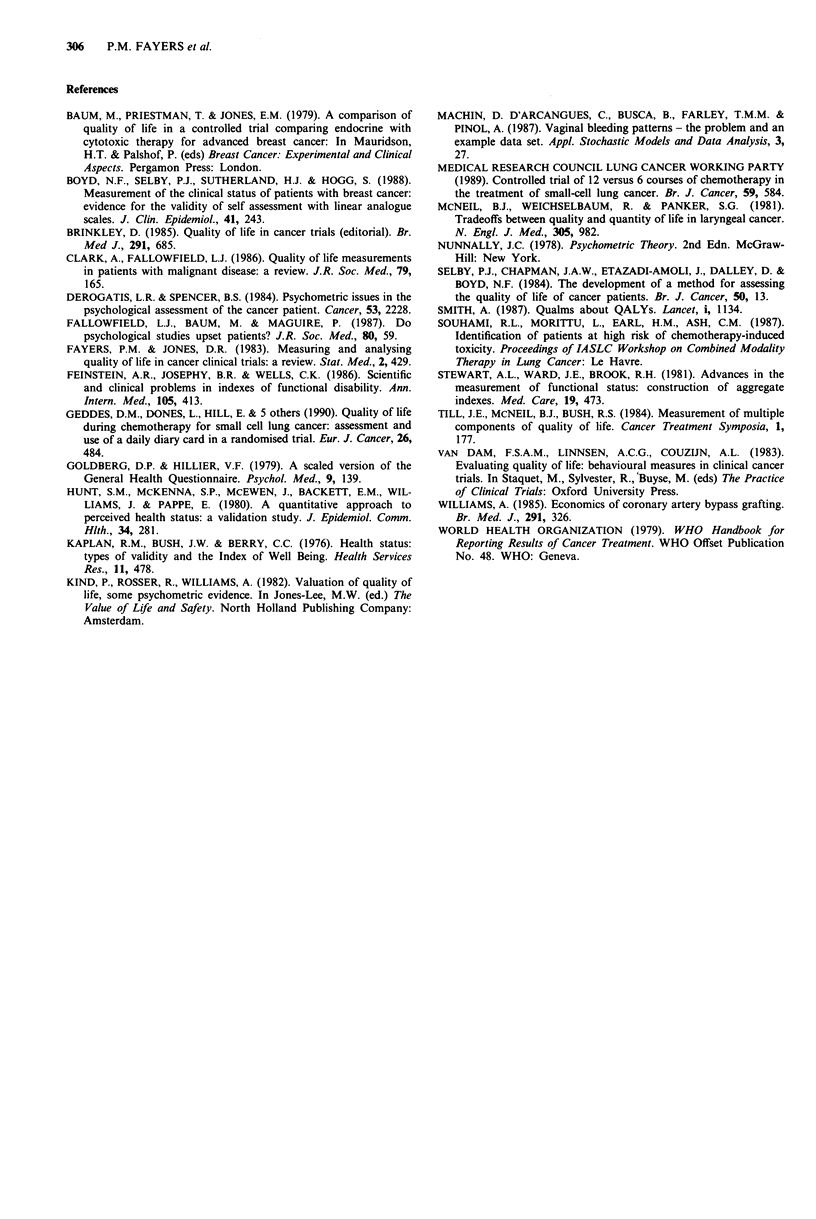


## References

[OCR_00988] Boyd N. F., Selby P. J., Sutherland H. J., Hogg S. (1988). Measurement of the clinical status of patients with breast cancer: evidence for the validity of self assessment with linear analogue scales.. J Clin Epidemiol.

[OCR_00994] Brinkley D. (1985). Quality of life in cancer trials.. Br Med J (Clin Res Ed).

[OCR_00998] Clark A., Fallowfield L. J. (1986). Quality of life measurements in patients with malignant disease: a review.. J R Soc Med.

[OCR_01003] Derogatis L. R., Spencer P. M. (1984). Psychometric issues in the psychological assessment of the cancer patient.. Cancer.

[OCR_01006] Fallowfield L. J., Baum M., Maguire G. P. (1987). Do psychological studies upset patients?. J R Soc Med.

[OCR_01010] Fayers P. M., Jones D. R. (1983). Measuring and analysing quality of life in cancer clinical trials: a review.. Stat Med.

[OCR_01013] Feinstein A. R., Josephy B. R., Wells C. K. (1986). Scientific and clinical problems in indexes of functional disability.. Ann Intern Med.

[OCR_01018] Geddes D. M., Dones L., Hill E., Law K., Harper P. G., Spiro S. G., Tobias J. S., Souhami R. L. (1990). Quality of life during chemotherapy for small cell lung cancer: assessment and use of a daily diary card in a randomized trial.. Eur J Cancer.

[OCR_01024] Goldberg D. P., Hillier V. F. (1979). A scaled version of the General Health Questionnaire.. Psychol Med.

[OCR_01034] Kaplan R. M., Bush J. W., Berry C. C. (1976). Health status: types of validity and the index of well-being.. Health Serv Res.

[OCR_01055] McNeil B. J., Weichselbaum R., Pauker S. G. (1981). Speech and survival: tradeoffs between quality and quantity of life in laryngeal cancer.. N Engl J Med.

[OCR_01064] Selby P. J., Chapman J. A., Etazadi-Amoli J., Dalley D., Boyd N. F. (1984). The development of a method for assessing the quality of life of cancer patients.. Br J Cancer.

[OCR_01068] Smith A. (1987). Qualms about QALYs.. Lancet.

[OCR_01076] Stewart A. L., Ware J. E., Brook R. H. (1981). Advances in the measurement of functional status: construction of aggregate indexes.. Med Care.

[OCR_01092] Williams A. (1985). Economics of coronary artery bypass grafting.. Br Med J (Clin Res Ed).

